# FleQ, FleN and c-di-GMP coordinately regulate cellulose production in *Pseudomonas syringae* pv. tomato DC3000

**DOI:** 10.3389/fmolb.2023.1155579

**Published:** 2023-03-27

**Authors:** Laura Martínez-Rodríguez, Aroa López-Sánchez, Andrea García-Alcaide, Fernando Govantes, María-Trinidad Gallegos

**Affiliations:** ^1^ Department of Soil and Plant Microbiology, Granada, Spain; ^2^ Centro Andaluz de Biología del Desarrollo, Universidad Pablo de Olavide/Consejo Superior de Investigaciones Científicas/Junta de Andalucía, Sevilla, Spain; ^3^ Departamento de Biología Molecular e Ingeniería Bioquímica, Universidad Pablo de Olavide, Sevilla, Spain

**Keywords:** FleN, FleQ, c-di-GMP, *Pseudomonas syringae*, cellulose, transcriptional regulation

## Abstract

The second messenger cyclic di-GMP (c-di-GMP) controls the transition between motility and sessility in many bacterial species by a variety of mechanisms, including the production of multiple exopolysaccharides. *Pseudomonas syringae* pv. tomato (Pto) DC3000 is a plant pathogenic bacteria able to synthesize acetylated cellulose under high c-di-GMP levels thanks to the expression of the *wssABCDEFGHI* operon. Increased cellulose production enhances air-liquid biofilm formation and generates a wrinkled colony phenotype on solid media. We previously showed that under low levels of c-di-GMP, the regulators FleQ and AmrZ bound to adjacent sequences at the *wss* promoter inhibiting its expression, but only FleQ responded to the presence of c-di-GMP by activating cellulose production. In the present work, we advance in the knowledge of this complex regulation in Pto DC3000 by shedding light over the role of FleN in this process. The distinctive features of this system are that FleN and FleQ are both required for repression and activation of the *wss* operon under low and high c-di-GMP levels, respectively. We have also identified three putative FleQ binding sites at the *wss* promoter and show that FleQ/FleN-ATP binds at those sites under low c-di-GMP levels, inducing a distortion of DNA, impairing RNA polymerase binding, and repressing *wss* transcription. However, binding of c-di-GMP induces a conformational change in the FleQ/FleN-ATP complex, which relieves the DNA distortion, allows promoter access to the RNA polymerase, and leads to activation of *wss* transcription. On the other hand, AmrZ is always bound at the *wss* promoter limiting its expression independently of FleQ, FleN and c-di-GMP levels.

## Introduction

*Pseudomonas syringae* pv. tomato (Pto) DC3000 is the causal agent of the disease known as bacterial speck on tomato (*Solanum lycopersicum*), whose symptoms are necrotic lesions in the aerial parts of the plant. It also infects *Arabidopsis thaliana* and several *Brassica* species, and causes a hypersensitive response (HR) in incompatible hosts such as tobacco (*Nicotiana tabacum*), *Nicotiana bethamiana* or bean (*Phaseolus vulgaris*) (Whalen et al., 1991; Preston, 2000; Zhao et al., 2000; Fouts et al., 2003). Like most *P. syringae* pathovars, Pto DC3000 penetrates into the leaves through wounds or stomata. However, it is a weak epiphyte and does not proliferate on the leaf surface, therefore depending on a rapid entry to the intercellular space (apoplast), where it actively multiplies (Boureau et al., 2002; Melotto et al., 2008; Xin and He, 2013; Nogales et al., 2015). Its main virulence factors are a collection of effectors that are secreted through a type III secretion system (T3SS) and a polyketide phytotoxin called coronatine, which mimics the plant growth regulator methyl jasmonate (Xin et al., 2018). In addition, Pto DC3000 has other mechanisms that contribute to the success of the infection: different types of motility, which allow escape from hostile environments and entry through stomata during the infection process; the production of biosurfactants, which participate in motility and resistance to adverse conditions; or exopolysaccharides (EPS), which prevent desiccation. Motility mostly relies on the presence of flagella, but these organelles also play important roles in surface adhesion, secretion and biofilm formation (Chaban et al., 2015). Flagella are essential for swimming, which takes place individually in liquid or viscous media, and for swarming, a synchronized movement of a population on solid or semi-solid surfaces. In addition to the presence of functional flagella, swarming requires the production of biosurfactants that act by reducing surface tension and facilitating bacterial movement (Kearns, 2010). Pto DC3000 produces several polar flagella and an array of linear lipopeptides called syringafactins. They are synthesized by a non-ribosomal peptide synthetase encoded by two genes (*syfA* and *syfB*) and are absolutely necessary for Pto DC3000 swarming, since a *syfA* mutant is unable to move on surfaces (Berti et al., 2007; Nogales et al., 2015). Regarding EPSs, Pto DC3000 is able to synthesize alginate, levans, acetylated cellulose and a Psl-like EPS (Pérez-Mendoza et al., 2014; Heredia-Ponce et al., 2020).

Bis-(3’-5’)-cyclic diguanosine monophosphate, also called cyclic diguanylate or c-di-GMP, is a ubiquitous second messenger in bacteria. The intracellular concentration of this nucleotide varies with multiple environmental signals and plays an essential role in regulating the transition from a free and motile lifestyle to a sessile one, in association with eukaryotic hosts and/or forming biofilms. The most captivating feature of c-di-GMP signalling is that it carries out its regulation at multiple levels: transcriptional, post-transcriptional and post-translational, by binding to different effector molecules (Römling et al., 2013). One of these effectors is the transcriptional regulator FleQ from *Pseudomonas* (FlrA in *Vibrio* spp.), an activator of the RNA polymerase with the sigma factor RpoN (σ54) that binds and hydrolyses ATP, but also responds to variations in the intracellular concentrations of c-di-GMP by modifying its activity (Baraquet et al., 2012; Srivastava et al., 2013). FleQ belongs to the bacterial enhancer binding protein (bEBP) family which possess three distinctive domains: a REC domain at its N-terminus, a central AAA+ domain with ATPase activity that also interacts with σ54, and a helix-turn-helix domain for DNA binding at its C-terminus (Arora et al., 1997; Baraquet and Harwood, 2013; Srivastava et al., 2013; Matsuyama et al., 2016).

As a bEBP, FleQ has a well-known role as master regulator of *Pseudomonas* flagella gene expression since, together with σ54, is essential for the transcriptional activation of class II genes, ultimately leading to FliC (flagellin) synthesis and flagella production. Interestingly, FleQ not only binds to enhancer sequences upstream of the transcription initiation site, like classic bEBPs, but also at sites downstream from the transcription initiation point (Arora et al., 1997; Jyot et al., 2002; Dasgupta et al., 2003; Baraquet et al., 2012; Leal-Morales et al., 2022). FleQ activity on the class II flagellar promoters is antagonized by c-di-GMP in *P. aeruginosa* and *P. putida*. c-di-GMP binds to FleQ at a site contiguous to the ATP binding pocket, causing the displacement of the N-terminus of the protein towards the ATP binding site, which allosterically inhibits its ATPase activity (Matsuyama et al., 2016; Banerjee et al., 2019). FleQ ATPase activity is required for the activation of σ^54^-dependent promoters, thus c-di-GMP downregulates flagellar gene expression (Hickman and Harwood, 2008; Starkey et al., 2009; Baraquet and Harwood, 2013; Nie et al., 2017; Navarrete et al., 2019). In addition, FleQ controls surface attachment and biofilm formation in response to c-di-GMP, stimulating the synthesis of enzymes, regulators, adhesins, EPSs and other envelope components in a variety of *Pseudomonas* species (Baraquet et al., 2012; Mastropaolo et al., 2012; Baraquet and Harwood, 2016; Jiménez-Fernández et al., 2016; Matsuyama et al., 2016; Molina-Henares et al., 2017; Navarrete et al., 2019). However, the regulation of biofilm promoters is different from that of the flagellar genes. Transcription is not σ^54^-dependent, FleQ regulation may be positive, negative or a combination of both, and c-di-GMP always stimulates transcription, either by promoting activation or by antagonizing repression (Hickman and Harwood, 2008; Baraquet et al., 2012; Matsuyama et al., 2016; Navarrete et al., 2019). Therefore, FleQ is a c-di-GMP-responsive transcription factor that oppositely regulates the genes required for flagellar motility and surface adhesion in response to fluctuating intracellular levels of that second messenger.

Both flagellar and biofilm matrix genes are also under the control of another protein, FleN. The ATPase FleN and its orthologs (FlhG in *Vibrio, Campylobacter* and *Shewanella*) interact with the GTPase FlhF to determine the polar location and number of flagella in several bacteria (Kazmierczak and Hendrixson, 2013; Kojima et al., 2020). Deletion of *fleN/flhG* causes the upregulation of flagellar gene expression and a hyperflagellated phenotype (Dasgupta et al., 2000; Kusumoto et al., 2008). In *P. aeruginosa*, FleN also functions as an antiactivator of FleQ and, by interacting with it, reduces its ATPase activity and consequently the activation of flagellar genes (Dasgupta et al., 2000; Dasgupta and Ramphal, 2001; Baraquet and Harwood, 2016; Chanchal et al., 2017). It has been shown that, although FleN-ATP modifies the structure of FleQ, it does not hinder the binding of FleQ to DNA (Dasgupta and Ramphal, 2001). Apart from inhibiting the transcriptional activation of FleQ-dependent flagellar genes, FleN participates in the regulation of FleQ-dependent biofilm matrix genes in *P. aeruginosa* and *P. putida* by assisting FleQ binding and/or repositioning on the target promoters (Hickman and Harwood, 2008; Baraquet et al., 2012; Baraquet and Harwood, 2016; Matsuyama et al., 2016; Navarrete et al., 2019). Deletion of *fleN* caused decreased expression of biofilm matrix genes, such as *pelA-F* (Pel EPS), *pslA-L* (Psl EPS), *cdrAB* (an adhesin) and PA2440 (coding for a polysaccharide deacetylase) in *P. aeruginosa* (Hickman and Harwood, 2008; Baraquet and Harwood, 2016). In *P. putida fleN* deletion decreased the transcription of *lapA* (a large adhesin), but increased that of the *bcs* (cellulose production) operon (Nie et al., 2017; Navarrete et al., 2019). In summary, c-di-GMP and ATP-bound FleN interact with FleQ to inhibit its ATPase activity and, although the mechanism and the effect on transcription varies according to the promoter, all three coordinate the regulation of flagellar motility and biofilm development, promoting a sessile state of life to the detriment of a motile one, or *vice versa*.

As in other bacteria, the artificial increase of intracellular c-di-GMP levels in Pto DC3000 inhibited motility but promoted cellulose and alginate production, improved pellicle formation under aerobic and static conditions, and led to a colony morphotype similar to the rdar (red, dry, and rough) or WS (Wrinkly Spreader) (Pérez-Mendoza et al., 2014; Farias et al., 2019). Increased intracellular c-di-GMP levels were achieved by overexpressing the heterologous diguanylate cyclase (DGC) PleD* from a stable plasmid vector (pJB3pleD*) in Pto DC3000 strains (Pérez-Mendoza et al., 2014). PleD* is a well-characterized mutant variant of the *Caulobacter crescentus* PleD protein which carries four point mutations that generate a constitutively active DGC, regardless of its phosphorylation status (Aldridge et al., 2003).

Cellulose production in Pto DC3000 is a complex process controlled at multiple levels. In addition to being directly post-translationally regulated by c-di-GMP (this second messenger allosterically activates WssB cellulose synthase by binding to its PilZ domain), is also transcriptionally regulated by two independent transcriptional regulators, FleQ and AmrZ. AmrZ (alginate and motility regulator Z) is a highly conserved transcriptional regulator within the pseudomonads. Initially described as the regulator of the alginate operon (Baynham et al., 1999), it has been characterized as a global regulator essential for environmental sensing and adaptation in *P. aeruginosa* and *P. ogarae* F113, where it acts as transcriptional activator and/or repressor of numerous genes (Jones et al., 2014; Martínez-Granero et al., 2014; Blanco-Romero et al., 2022). In Pto DC3000 AmrZ also acts as a bifunctional regulator, repressing cellulose production and activating alginate production, motility and virulence (Prada-Ramírez et al., 2016). Regarding cellulose production, both FleQ and AmrZ bind directly to the promoter region of the *wssABCDEFGHI* biosynthetic operon inhibiting its expression under physiological c-di-GMP levels. However, their behaviours are completely different under high c-di-GMP. AmrZ represses the transcription of the *wss* operon independently of c-di-GMP, whereas FleQ converts from a repressor to an activator upon c-di-GMP binding (Pérez-Mendoza et al., 2019). Since FleN has an important role in FleQ-dependent regulation of both flagellar genes and biofilm matrix coding operons in *P. aeruginosa* and *P. putida* (Hickman and Harwood, 2008; Baraquet et al., 2012; Baraquet and Harwood, 2016; Matsuyama et al., 2016; Navarrete et al., 2019), our aim was to shed light over the possible interaction of AmrZ, FleQ, c-di-GMP and FleN at the Pto DC3000 *wss* promoter.

## Materials and methods

### Bacterial strains and growth conditions

The bacterial strains used in this study are listed in Table 1. *E. coli* and *P. syringae* pv. tomato DC3000 strains were routinely grown in Luria-Bertani (LB) medium (Sambrook et al., 1989) at 28°C. Pto DC3000 was also grown in MMR (7 mM Na-glutamate, 55 mM mannitol, 1.31 mM K_2_HPO_4_, 2.2 mM KH_2_PO_4_, 0.61 mM MgSO_4_, 0.34 mM CaCl_2_, 0.022 mM FeCl_3_, 0.85 mM NaCl) minimal medium (Robertsen et al., 1981) at 20°C. When required, other compounds like antibiotics were added: gentamicin (2–10 μg/ml), kanamycin (25 μg/ml), rifampicin (10 μg/ml) and tetracycline (10 μg/ml).

**TABLE 1:** Bacterial strains and plasmids used.

Strain or plasmid	Relevant characteristics	References
**Strains**		
*P. syringae* pv. tomato		
DC3000	Wild type; Rif^R^	Cuppels (1986)
*fleQ*	Δ*fleQ*::ΩKm; Rif^R^ Km^R^	Vargas et al. (2013)
*fleN*	Δ*fleN*; Rif^R^	This work
*fliC*	*fliC (flaA)::miniTn5Cm*; Rif^R^ Cm^R^	Hu et al. (2001)
*wssBC*	*ΔwssBC*; Rif^R^	Pérez-Mendoza et al. (2014)
*E. coli*		
One Shot BL21star (DE3)	F^−^ *ompT hsdS*_*B*_ (r_B_^−^, m_B_^−^) *gal dcm rne131* (DE3)	Thermo Fisher Scientific
**Plasmids**		
pBBRN	Gm^R^; pBBR1-MCS5 derivative cloning vector with an additional NdeI site	Fillet et al. (2009)
pBBRN::*fleN*	Gm^R^; pBBRN derivative with an 841 bp NdeI-SacI fragment containing the *fleN* gene	This work
pET28b(+)	Km^R^; protein expression vector, T7 promoter	Novagen
pET28*fleN*	Km^R^; pET28b(+) bearing a 841 bp NdeI-SacI fragment with *fleN*	This work
pET29Q	Km^R^; pET29a(+) bearing a 1481 bp NdeI/XhoI fragment with *fleQ*	Pérez-Mendoza et al. (2019)
pJB3Tc19	Ap^R^ Tc^R^; cloning vector, Plac promoter	Blatny et al. (1997)
pJB3pleD*	Ap^R^ Tc^R^; pJB3Tc19 bearing a 1423 bp XbaI/EcoRI fragment containing pleD*	Pérez-Mendoza et al. (2014)
pK18*mobsacB*	Km^R^; suicide vector	Schäfer et al. (1994)
pK18ΔfleN	Km^R^; pK18*mobsacB* with a 1629 pb EcoRI fragment bearing a deleted version of *fleN*	This work

Cm^R^, Gm^R^, Km^R^, Rif^R^, and Tc^R^ stand for resistance to chloramphenicol, gentamicin, kanamycin, rifampicin, and tetracycline, respectively.

EPS production can be detected and even quantified using dyes as calcofluor (CF) or Congo Red (CR). CR binds to neutral or basic polysaccharides and some proteins, whereas CF is more specific and binds to β(1–4) and β(1–3) glycosidic bonds, like those present in cellulose, and positive colonies fluoresce under UV light (Spiers et al., 2002). Colony morphology and EPS production were visualized on MMR plates with Congo Red (50 μg/ml) and calcofluor (50 μg/ml).

### Strain and plasmid construction

We generated the *ΔfleN* directed mutant by deleting most of its ORF (from nucleotide 10–816). First, a region with *fleN* adjacent sequences but lacking its ORF was amplified by PCR with specific oligonucleotides (Table 2) and cloned into pK18*mobsacB* (Schäfer et al., 1994), which does not replicate in *P. syringae*. The plasmid was then electroporated into Pto DC3000. Transformants were selected in kanamycin (50 μg/ml), screened for sucrose sensitivity (15% [w/v]), and then grown in liquid LB at 4°C to force plasmid loss. Cells were then plated on LB with sucrose (15% [w/v]) and the Suc^R^Km^S^ colonies, which were expected to be double-recombinants, were selected and check by PCR and sequencing.

**TABLE 2:** Oligonucleotides used in this work.

Name	Sequence (5’→3’)	
**Directed mutants**		
ΔfleN.1	aaagaa​ttcGGA​AGA​GGG​CGG​AGT​GAT​TG	
ΔfleN.2	GCA​CAG​GCC​CCG​CGC​TGC​C**CAT**GTT​ATT​TC	
ΔfleN.3	GAA​ATA​AC**ATG**GGC​AGC​GCG​GGG​CCT​GTG​C	
ΔfleN.4	aaagaa​ttCCTG​CTA​CCA​CGT​AAC​GC	
**Plasmid construction**		
fleN_NdeI	aaa​aacat **ATG** GGC​AGC​ATG​CAT​CCC​G	
fleN_SacI	tat​ttagag​c **tcaTC​A**TTG​CAC​AGG​CCC​CGC	
**PCR for *in vitro* assays**		
wssA-F	CCA​GCC​ACT​GAT​TTA​ATT​CG	Amplify a 323 bp fragment (wssA1-2) upstream *wssA* CDS, between nucleotides 1119694 and 1120134
wssA2-R2	TGGTTGCTCGATAGACGG	
**RT-qPCR**		
gyrA+	GGC​AAG​GTC​ACC​CGC​TTC​AAG​GAA​T	Amplify a 127 bp fragment of *gyrA*
gyrA-	GAC​CGC​CAC​GCT​TGT​ACT​CAG​GGA​AC	
wssB+	GGT​GTT​CAA​CGC​TGT​GAC​GCA​GGA	Amplify a 198 bp fragment of *wssB*
wssB-	TGG​CGC​AGT​GAA​AGA​TCA​TCG​AAA​CG	

Capital letters: match to the sequence; lower case: added sequence; underlined: newly created restriction sites; bold: start or stop codon.

We constructed the pET28*fleN* plasmid for His-FleN overexpression and purification. The *fleN* gene was cloned into pET28b(+) as a NdeI-SacI fragment after PCR of chromosomal DNA with the oligonucleotides fleN-NdeI and fleN-SacI (Table 2). We also constructed a plasmid bearing the *fleN* gene for *in trans* complementation of the respective deficient mutant by digesting the pET28*fleN* plasmid with NdeI and SacI and ligating the fragment into pBBRN (Fillet et al., 2009) digested with the same enzymes.

Plasmid transformation of Pto DC3000 strains were carried out by electroporation. Electro-competent cells were prepared according to Choi et al. (2006), mixed with DNA (0.3–0.5 μg of DNA per ml of cell suspension) in 0.1 cm cuvettes and electroporated with a high-voltage pulse (1800 V) for 5 ms by using an Eppendorf electroporator 2510. Transformants were selected in LB agar plates supplemented with the appropriate antibiotics.

### Biofilm development and quantification

#### Solid-liquid (S-L) biofilm formation

Pto DC3000 and mutants were grown on LB plates for 48 h, inoculated into 3 ml of LB with rifampicin (10 μg/ml) and grown for 24 h. 150 μL of cultures adjusted to an A_660_ of 0.1 in MMR with 2 mM CaCl_2_ were dispensed into wells of Costar 96 microtiter polystyrene plates (Corning). The plates were incubated at 20°C with moderate shaking (150 rpm) for the desired period of time and processed for planktonic and biofilm growth quantification, as described before (O'Toole et al., 1999). Serial dilution-based growth curves were performed as previously described (López-Sánchez et al., 2013). For each experiment, at least 3 biological replicates were assayed in octuplicate.

#### Air-liquid (A-L) biofilm or pellicle formation

For pellicle detection, Pto DC3000 and mutants were grown on LB plates for 48 h and resuspended in sterile milliQ water. 2 ml of cultures adjusted to an A_660_ of 0.05 in MMR were dispensed into wells of Nunclon Delta surface 24 microtiter polycarbonate plates (Nunc) and incubated at 20°C under aerobic and static conditions for 72 h. The appearance of the bacterial communities at macroscopic level was studied after taking photographs directly from the plates.

### Quantification of cellulose production

Calcofluor binding assays by the different strains were performed as follows: bacteria were suspended from fresh LB plates in sterile milliQ water, diluted into 10 ml flasks containing MMR supplemented with CF (50 μg/ml final concentration) to an initial A_660_ of 0.05, and incubated at 20°C under agitation for 24 h. Cultures were then centrifuged for 15 min at 4000 rpm, supernatant containing unbound CF broth was removed and the pellet was then suspended in 10 ml of distilled water. CF binding measurements for 6 biological replicates of each strain were performed in a PTI fluorimeter (Photon Technology International), after confirming a similar growth of all strains, and expressing the results in arbitrary units ± standard deviation.

### Protein purification

AmrZ and FleQ were purified as described before (Prada-Ramírez et al., 2016; Pérez-Mendoza et al., 2019). For FleN purification, the One Shot BL21star (DE3) (pET28b(+):*fleN*) cells were grown at 28°C in 2-L Erlenmeyer flasks containing 1 L of 2 × YT culture medium (Sambrook et al., 1989) supplemented with kanamycin (50 μg/ml). Protein expression was induced at an A_660_ of 0.2–0.3 by adding 0.5 mM isopropyl β-D-1-thiogalactopyranoside and cultures were grown for another 5 h at 15°C, when they were harvested by centrifugation at 5000 × *g*. The pellet resulting from a 500 ml culture was resuspended in 25 ml of buffer A (25 mM Na-phosphate pH 7.0, 500 mM NaCl, 5% glycerol) with protease inhibitor mixture (Complete™, Roche) and broken by treatment with 20 μg/ml of lysozyme and French press. Following centrifugation at 13,000 × *g* for 60 min, the FleN protein was predominantly present in the soluble fraction. The supernatant was loaded onto a 5 ml Hi-Trap chelating column (GE Healthcare), equilibrated with buffer A and eluted with a gradient of a 50 mM-1 M imidazole. Fractions containing His_6_-FleN were pooled and was dialyzed against 20 mM Tris-HCl pH 8.0, 500 mM NaCl, 10% glycerol and stored at −80°C. Protein concentrations were determined using the Bio-Rad Protein Assay kit.

#### Electrophoretic mobility shift assays (EMSA)

A 458 bp fragment containing the *wssA* promoter region (wssA1-2) obtained from DC3000 chromosomal DNA by PCR was used as DNA probe (Table 2). The PCR product was isolated from an agarose gel by using the Nucleospin gel and PCR clean-up (Macherey-Nagel) and radiolabelled at its 5’-ends with [γ-^32^P]ATP and T4 polynucleotide kinase. The labelled probe (20 nM) was then incubated with the indicated concentrations of purified FleQ, FleN and/or AmrZ in 10 μL of STAD (25 mM Tris-acetate pH 8.0, 8 mM Mg-acetate, 10 mM KCl, 3.5% (w/v) polyethylene glycol-8000 and 1 mM DTT) supplemented with 10 μg/ml of poly(dI-dC), and 200 μg/ml of bovine serum albumin. The reaction mixtures were incubated for 30 min at 4°C, and samples were run on 4% (w/v) native polyacrylamide gels (Bio-Rad Mini-Protean) for 2 h at 50 V at room temperature in Tris-glycine (25 mM Tris, 200 mM glycine). The results were analysed with Personal FX equipment and Quantity One software (Bio-Rad).

### DNA footprints

The DNA probe was the 458-bp wssA1-2 PCR fragment containing the *wssA* promoter region (Prada-Ramírez et al., 2016; Pérez-Mendoza et al., 2019). For the footprint on the top strand, the PCR was carried out with primers wssA-F [end labeled with [γ-^32^P]ATP as described above] and wssA2-R. For the footprint on the bottom strand, the same primers were used, but in this case, wssA2-R was end-labeled. Purified labeled probe (20 nM) was incubated without or with FleQ (0.5 µM), His-FleN (0.5 µM), AmrZ (0.5 µM), ATP, c-di-GMP and/or c-di-AMP (0.5 mM) in 50 μL reaction volume of STAD (25 mM Tris-acetate pH 8.0, 8 mM Mg-acetate, 10 mM KCl, 3.5% (w/v) polyethylene glycol-8000 and 1 mM DTT) supplemented with 10 μg/ml of poly(dI-dC), and 200 μg/ml of bovine serum albumin. Reaction mixtures were incubated for 30 min at 4°C before being treated with DNase I or DMS, as described previously (Rojas et al., 2003; Guazzaroni et al., 2004). The results were analysed with Personal FX equipment and Quantity One software (Bio-Rad).

### *In vitro* transcription assays

Reactions (10 μL) were performed in STA buffer (25 mM Tris-acetate pH 8.0, 8 mM Mg-acetate, 10 mM KCl, 1 mM DTT and 3.5% (w/v) PEG 8000) with 0.5 μM FleQ, 0.5 µM His-FleN, 100 nM AmrZ, 0.5 mM nucleotides, 4 Units of RNAse inhibitor (Roche) and 5 nM DNA template (wssA1-2 PCR fragment). After 30 min incubation at 4°C, 0.5 U σ^70^-holoenzyme (New England Biolabs) were added and the reactions were incubated for 5 min at 30°C before the addition of 1.2 μL of the following elongation mixture: 0.1 mM each for ATP, CTP and GTP, 0.05 mM UTP and 50 μCi [α-^32^P]UTP. After a further 15 min incubation at 30°C, the reactions were stopped by adding 3.7 μL of formamide sequencing dye. Samples were electrophoresed in a 6.5% (w/v) polyacrylamide denaturing sequencing gel. The results were analysed with Personal FX equipment and Quantity One software (Bio-Rad).

### Statistical analysis

Statistical treatment of data was performed using R or Graphpad Prism 6 software. Comparison among different strains or conditions was performed by one-way ANOVA with *post hoc* Tukey HSD test.

## Results

### Role of *fleN* in biofilm development

**FIGURE 1:**
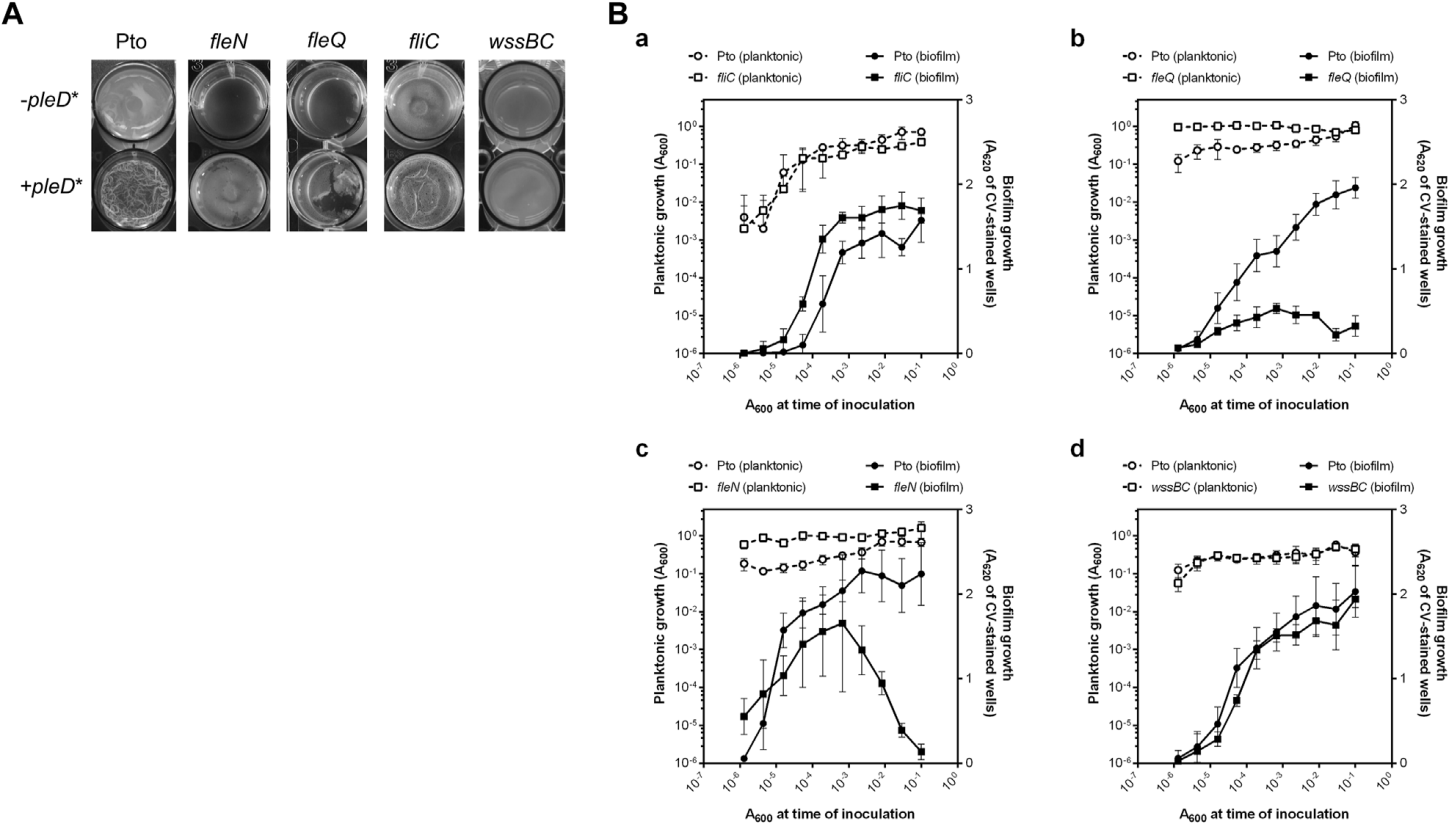
Biofilm development by Pto DC3000 mutant strains. **(A)** Air-liquid biofilm formation. Pto and the *fliC*, *fleQ*, *fleN* and *wssBC* mutants were grown under aerobic and static conditions at 20°C in 24-multiwell plates for 72 h in MMR supplemented with tetracycline (10 μg/ml), when images were directly taken from the plate. **(B)** Solid-liquid biofilm formation. Serial dilution-based growth curves of the indicated strains where planktonic (left axes, open symbols) or biofilm growth (right axes, closed symbols) is plotted against the initial A_600_ of each dilution. Circles represent the wild type strain and squares represent the *fliC* **(a)**, the *fleQ* **(b)**, the *fleN* **(c)** and the *wssB* **(d)** mutants. Plots display one representative experiment of at least three biological replicates. Error bars represent the standard deviation of the eight technical replicates.

We previously showed that the artificial increase of the c-di-GMP intracellular levels by the overexpression of the heterologous diguanylate cyclase PleD* in Pto DC3000 stimulated the formation of biofilms that are associated with the air-liquid (A-L) interface, also called pellicles (Pérez-Mendoza et al., 2014; Farias et al., 2019). Pto DC3000 mature pellicles are white, translucent and adhere slightly to the walls of the container in which they are grown, but quickly sink if the container is moved. In the absence of pleD* (pJB3Tc19) Pto also forms pellicles, but they are weaker, more transparent, and not wrinkled (Figure 1A).

To study the *in vivo* effect of FleN, we constructed a *fleN* mutant and a plasmid with the intact gene for *in trans* expression. First, pellicle formation by the *fleQ*, *fleN*, and *fliC* mutant strains was evaluated under normal and artificially increased c-di-GMP levels (Figure 1A). In the absence of pleD* all the mutant strains formed pellicles in aerobic and static conditions and, similarly to those of the wild type, they were thin, transparent and not wrinkled. The artificial increase of c-di-GMP intracellular levels by PleD* facilitated the formation of pellicles in all of the mutants assayed, however, only those of the *fleN* mutant were similar to the wild type. The pellicles of the *fleQ* and *fliC* mutants, which lack flagella (Vargas et al., 2013; Nogales et al., 2015), were very different from the pellicles generated by the wild type and *fleN* mutant, and were often broken and disintegrated in flocs (Figure 1A). As shown before, the mutant *wssBC*, which does not produce cellulose, also formed pellicles at high intracellular levels of c-di-GMP, but its appearance and consistency were different (Pérez-Mendoza et al., 2014; Farias et al., 2019). The wild type and all the tested mutants except *wssBC*, fluoresced in the presence of pleD* when stained with calcofluor white, exhibiting an extracellular matrix formed by cellulose fibres (Supplementary Figure S1). Therefore, the matrix of these biofilms is mainly composed of cellulose which give them a characteristic opaque and wrinkled appearance.

Pto DC3000 is also able to produce solid-liquid (S-L) biofilms in the presence of CaCl_2_ (Fishman et al., 2018). For that reason, S-L biofilm formation by the wild type and *fleQ*, *fleN*, and *fliC* mutant strains was assessed by means of serial dilution-based growth curves in a culture medium with 2 mM CaCl_2_ (López-Sánchez et al., 2013). The wild type formed a biofilm upon entering the stationary phase, and no biofilm dispersion occurred for the duration of the assay (Figure 1B). A similar behaviour was observed with the *fliC* mutant [Figure 1B(a)]. In contrast, the *fleQ* mutant was severely impaired in biofilm formation [Figure 1B(a)] and the *fleN* mutant displayed premature biofilm dispersal compared to the wild type and produced less biofilm [Figure 1B(c)]. These results strongly suggest that functional flagella are not required for S-L biofilm formation, but FleQ is a major player in biofilm development. FleN is not needed for S-L biofilm formation but seems to have a role preventing biofilm dispersal. In order to evaluate the role of cellulose in this type of biofilm, we also analysed the behaviour of the *wssBC* mutant observing that it was almost identical to the wild type (Figure 1B(d)). Therefore, cellulose does not seem to be required for S-L biofilm development, unlike what occurs in pellicles.

## Role of *fleN* in colony morphology and cellulose production

**FIGURE 2:**
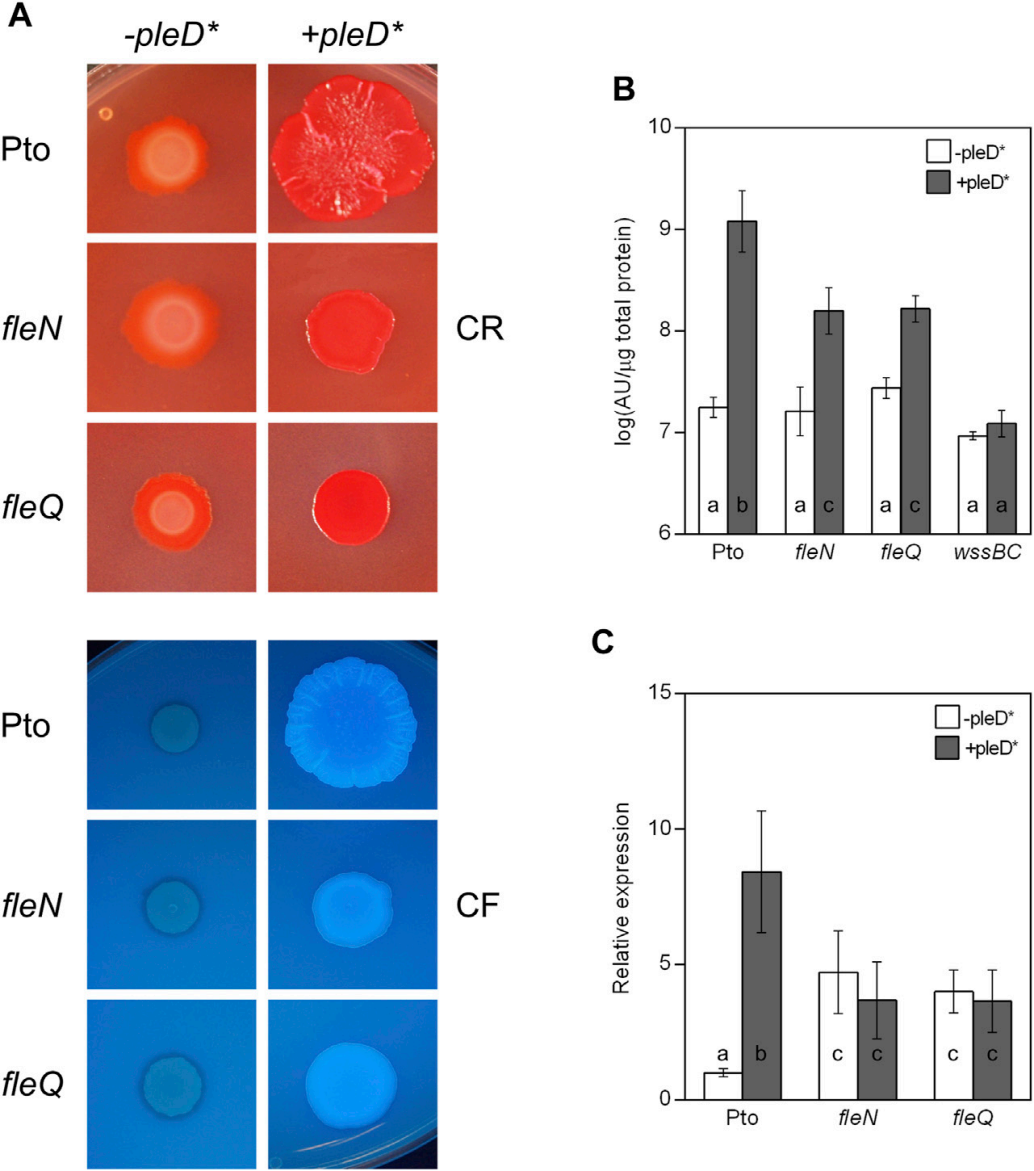
Colony morphology and cellulose production in the *fleN* mutant. **(A)** Colony morphology of the fleN mutant. Representative colony morphology of the different strains grown in agar plates supplemented with Congo Red and Calcofluor in the presence and in the absence of pleD*. Five μl of bacterial suspensions at A_660_ = 1.0 were placed on the surface of MMR plates with CR (50 μg/ml, top) or CF (100 μg/ml, bottom) and pictured after incubation at 20°C for 3 days and then at 10°C for 5 days. **(B)** Cellulose production at different c-di-GMP levels. Pto and the *fleN* and *fleQ* mutants were grown in MMR with CF (100 μg/ml) for 24 h at 20°C, and the fluorescence emission of the cell attached CF in liquid cultures was measured. The graphs show the average amount of cellulose produced by the indicated strains in the absence (white bars) and in the presence of pleD* (grey bars) as fluorescence (in arbitrary units) referred to total cell protein. Note that the *wssBC* mutant does not produce cellulose, therefore the bars indicate non-specific CF retention. Error bars correspond to the standard deviation of three biological replicates and a-c denote ANOVA categories with significant differences (*p* < 0.01). **(C)** Effect of c-di-GMP on the expression of the wss operon. Total RNAs were obtained from bacteria grown in MMR at 20°C for 24 h. Results show qRT-PCR of *wssB* in the wild type strain (Pto) and *fleN* and *fleQ* mutants, with pJB3Tc19 (in the absence of pleD*, white bars) or with pJB3pleD* (in the presence of pleD*, grey bars). Expression values were normalised with the housekeeping gene *gyrA* and referred to the wild type condition in the absence of pleD*. The graph shows the average mRNA levels and error bars correspond to the standard deviation of four biological replicates; a-c denote ANOVA categories with significant differences (*p* < 0.01).

The colony morphology of the *fleN* mutant was observed on plates with Congo red (CR) and calcofluor (CF), both in the absence and in the presence of pleD* (Figure 2A). At physiological c-di-GMP levels (i.e., in the absence of pleD*), the *fleN* mutant produced colonies similar to those of the wild type: pink on plates supplemented with CR and non-fluorescent under UV light on CF plates. At high levels of c-di-GMP (i.e., in the presence of pleD*) the mutant formed slightly rough colonies that were red on CR and fluorescent on CF, instead of the red/bright rosettes observed with the wild type. The *in trans* expression of *fleN* altered the aspect of the wild type and the *fleN* and *fleQ* mutant colonies. They became redder in CR and more fluorescent in CF in the absence of pleD*, but their appearance did not significantly change in the presence of pleD* (Supplementary Figure S2). The fact that expressing *fleN* from a plasmid makes the colonies redder and slightly more fluorescent at low levels of c-di-GMP, suggests a pleiotropic effect of FleN overproduction that is independent of FleQ, but it may be related to flagellar assembly.

Next, cellulose production in liquid cultures was quantified observing no significant differences among the wild type, *fleN* and *fleQ* at physiological levels of c-di-GMP (Figure 2B). However, in the presence of pleD*, the *fleN* and *fleQ* mutants increased cellulose production, but those levels were 10% of those of the wild type overexpressing pleD*. In summary, regarding cellulose production, the phenotype of the *fleN* mutant is very similar to that of the *fleQ* mutant (Pérez-Mendoza et al., 2019). Overall, these results suggest that FleN has a positive role in the regulation of cellulose synthesis in Pto DC3000.

The expression of the *wssABCDEFGHI* operon for the synthesis and export of acetylated cellulose was previously shown to be negatively regulated by FleQ (Pérez-Mendoza et al., 2019). Since *P*. *aeruginosa*, and *P. putida* mutants devoid of FleN are affected in biofilm formation, and FleN acts as an auxiliary factor to FleQ regulation (Dasgupta and Ramphal, 2001; Hickman and Harwood, 2008; Baraquet et al., 2012; Baraquet and Harwood, 2013; Chanchal et al., 2017; Nie et al., 2017; Navarrete et al., 2019), we wondered whether FleN was involved in the transcriptional regulation of the *wss* operon in Pto DC3000. To test that, *wssB* gene expression was quantified by qRT-PCR in Pto DC3000, *fleN* and *fleQ*, both in the absence and in the presence of pleD* (Figure 2C). In the wild type, *wssB* mRNA levels were low in the absence of pleD* and increased in its presence. In the *fleQ* mutant, *wssB* expression increased in the absence of pleD* and remained the same in its presence, as shown before (Pérez-Mendoza et al., 2019). Interestingly, the expression of *wssB* in the *fleN* mutant was similar to the *fleQ* mutant (Figure 2C). These results indicate that FleQ and FleN are both negative regulators of the *wss* operon, and the positive effect of c-di-GMP on *wssB* mRNA levels is dependent on the presence of both FleQ and FleN. Therefore, FleQ seems to repress *wss* transcription under low c-di-GMP and activate it under high c-di-GMP levels and for both processes FleN is required.

## FleN and c-di-GMP modulate the interaction of FleQ with the *wss* promoter

**FIGURE 3:**
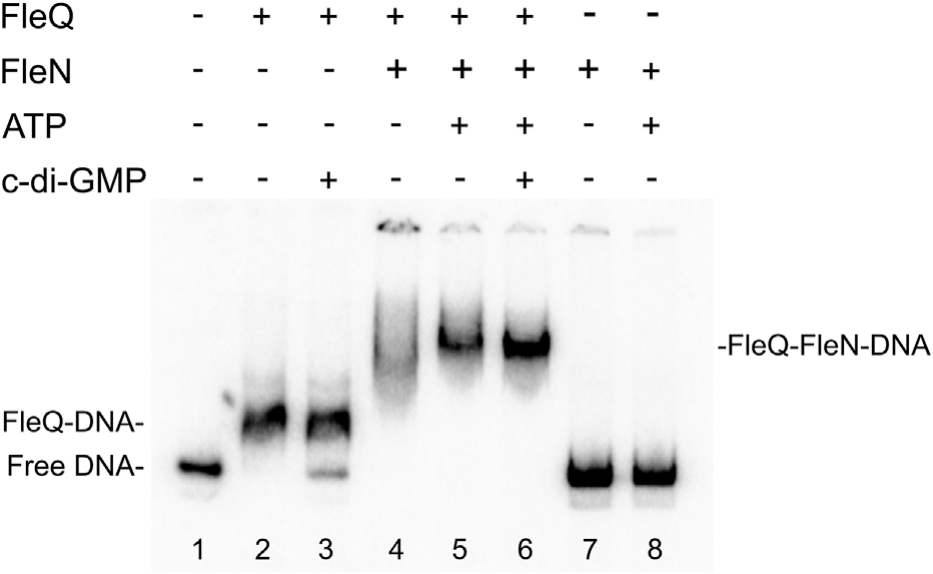
*In vitro* binding of the FleQ-FleN complex to the *wss* promoter region. Binding reactions were carried out as described in Materials and Methods with 1 nM of labelled wssA1-2 fragment in the absence (-) and in the presence of 1 μM FleQ, 1 μM FleN, 0.5 mM of ATP and 0.5 mM c-di-GMP. Putative shifted FleQ-DNA, and FleQ-FleN-DNA complexes are indicated.

To elucidate FleN mechanism of action at the *wss* promoter (P_*wssA*_) and its role in cellulose synthesis, Pto DC3000 FleN was overproduced heterologously in *E. coli* and purified as a N-terminal His_6_-FleN fusion. Interaction of FleQ and FleN with a P_*wssA*_ fragment was assessed *in vitro* by means of EMSA. Binding reactions in which each protein was added (separately or together) in a binding buffer with ATP and/or c-di-GMP were performed. In these conditions FleQ alone detectably retarded the probe (Figure 3 lane 2), as shown before (Pérez-Mendoza et al., 2019), whereas FleN alone, with or without ATP, did not so (Figure 3 lanes 7 and 8). In contrast, an equimolar mixture of FleQ and FleN resulted in a significantly retarded (but smeary) band compared with that of FleQ alone (Figure 3 lane 4). The addition of ATP to the reaction intensified that slower band (Figure 3 lane 5), indicating that FleN binds to FleQ to give higher-molecular-weight complexes and the ATP stabilizes the binding of the FleN-ATP/FleQ complex to DNA.

We showed before that c-di-GMP (but not c-di-AMP, ATP, or GTP) acts as an antagonist for FleQ repression at P_*wssA*_ facilitating the release of FleQ from the promoter DNA (Pérez-Mendoza et al., 2019). Here we observed that c-di-GMP partially disrupted the FleQ-DNA complex (Figure 3 lane 3) but not the FleQ/FleN-ATP/DNA complex (Figure 3 lane 6). Taken together, our EMSA results strongly suggest that FleQ binding to P_*wssA*_ is promoted by FleN, in a process that requires ATP.

**FIGURE 4:**
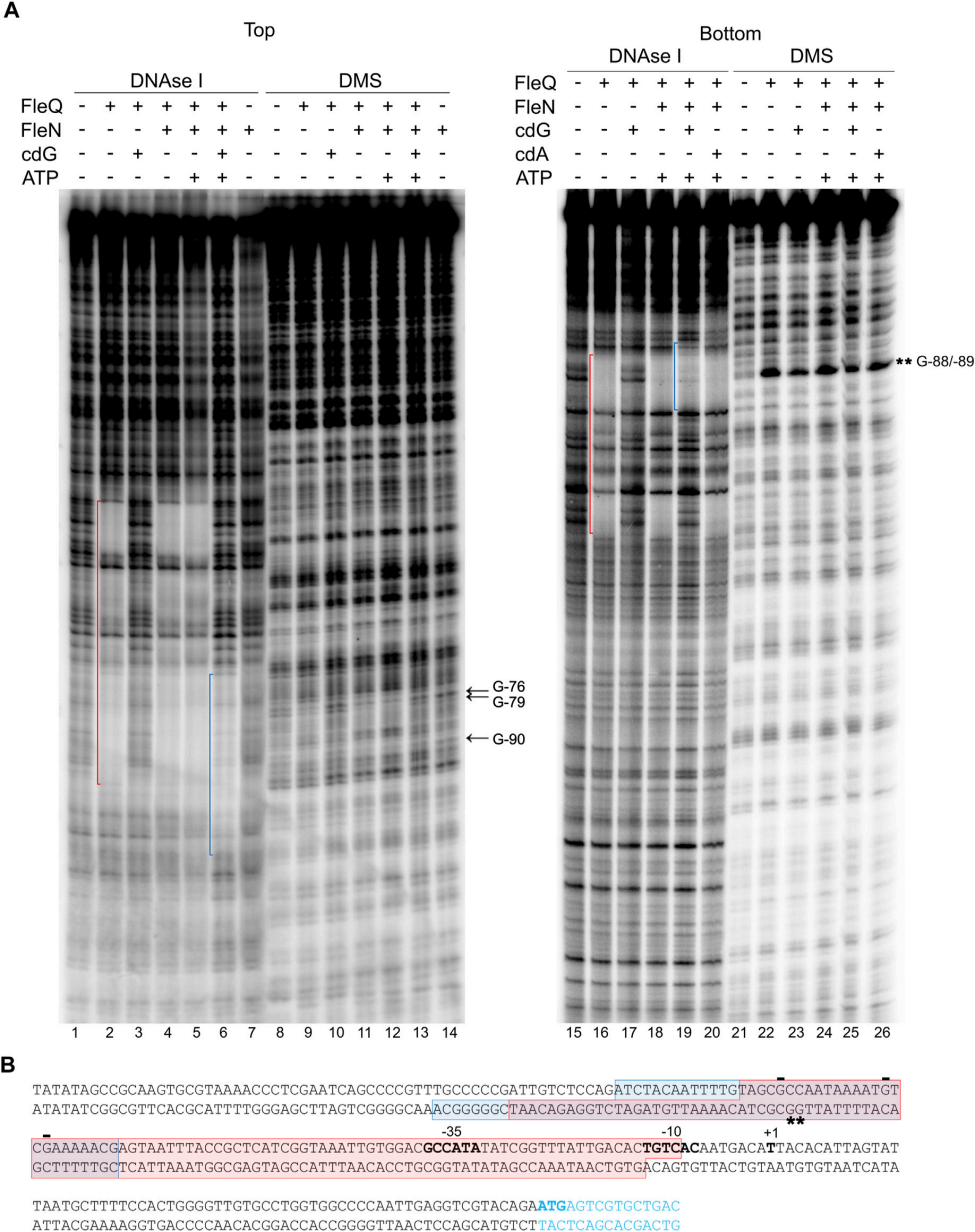
Identification of the FleQ-FleN complex binding site at the *wss* promoter by footprinting analysis. **(A)** DNAse I and DMS footprint. DNA probes corresponding to the *wssA* upstream region 5’ end-labeled on either the top or the bottom strand were prepared and incubated without (lanes -) and with FleQ (0.5 µm) and/or FleN (0.5 µm), ATP (0.25 mM) and c-di-GMP (0.25 mM). After partial digestion with DNase I or treatment with DMS and partial digestion with piperidine, the DNAs were subjected to urea-PAGE. Nucleotide sequences protected by FleQ and FleQ-FleN in the absence of c-di-GMP (red) or by the FleQ-FleN complex in the presence of c-di-GMP (blue) are indicated. Protected (arrow) and hyperreactive (*) nucleotides are also indicated. **(B)** Localisation of the FleQ and FleQ-FleN binding sites at the wss promoter. The boxes indicate the regions protected from DNAse I by FleQ or FleQ-FleN (red) and FleQ-FleN in the presence of c-di-GMP (blue) in the top and bottom strands. The -10 and -35 regions and the *wss* transcriptional start site are in bold. *, indicates hyperreactivity and -, protection.

DNase I footprinting was performed under the same conditions as the gel retardation assays to identify the location of the FleQ/FleN complex and binding sites at the *wss* promoter region (Figure 4). Interestingly, the DNase I protected region was a region of about 100 bp similar to that obtained with FleQ alone: from −94 to −10 at the top strand and from −118 to −13 at the bottom strand of the *wssA* upstream region, and the presence of ATP in the reaction did not modify it (Figure 4A, lanes 2, 4, 5, 16 and 18). However, this protection dramatically changed in the presence of c-di-GMP, moving upstream in the promoter from −69 to −107 at the top strand and from −69 to −126 at the bottom strand (Figure 4A, lanes 6 and 19 and Figure 4B). This is specific of c-di-GMP since the presence of c-di-AMP did not have any effect on the footprint (lane 20). As before, the protection against DNAse I conferred by FleQ alone was lost in the presence of c-di-GMP (lanes 3 and 17), suggesting that its interaction with the DNA has changed (Pérez-Mendoza et al., 2019). As in the EMSAs, FleN alone did not detectably bind to the DNA (lane 7).

To localize the FleQ/FleN-ATP binding sites accurately and to establish the guanosine residues in close contact with the bound complex, methylation protection patterns were determined on both DNA strands using dimethyl sulfate (DMS) as a footprinting reagent (Figure 4A). Protection of guanosine residues by FleQ/FleN-ATP was observed at the region identified by DNase I footprinting, thus confirming the location of the binding sites. Protection from DMS methylation was detected at G-76, G-79 and G-90 at the top strand when FleQ was bound to the DNA, either alone or in the presence of FleN (±ATP). G-76 and G-90 were protected in the absence of c-di-GMP (lanes 9, 11 and 12), but less in its presence (lanes 10 and 13). In the bottom strand, hyperreactivity at Gs -88 and -89 was observed when FleQ was present in the reaction (lanes 22–26), but diminished in the presence of c-di-GMP (lanes 23 and 25).

In summary, the binding of FleQ alone to the *wss* promoter is weakened in the presence of c-di-GMP. However, the FleQ/FleN-ATP complex remains bound even in the presence of c-di-GMP, but undergoes significant rearrangement, as shown by the alterations of DMS protection and hyperreactivity patterns, which indicate that the contacts have changed.

### Regulation of *wss* operon expression by FleQ, FleN, AmrZ and c-di-GMP

**FIGURE 5:**
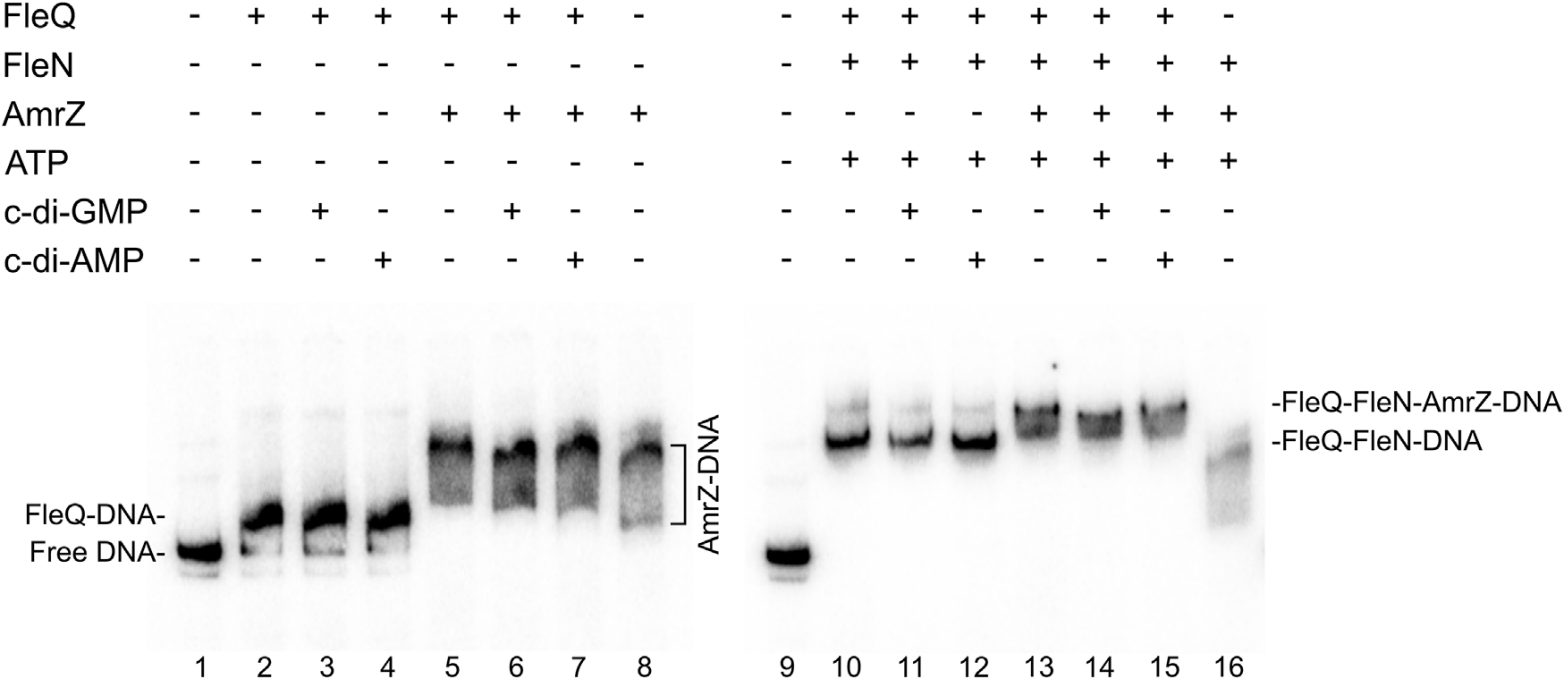
*In vitro* binding of the FleQ-FleN complex to the *wss* promoter region in the presence of AmrZ.Binding reactions were carried out in the absence (-) and in the presence (+) of FleQ, FleN, AmrZ and ATP, GTP, c-di-GMP or c-di-AMP. Putative shifted protein-DNA complexes are indicated.

**FIGURE 6:**
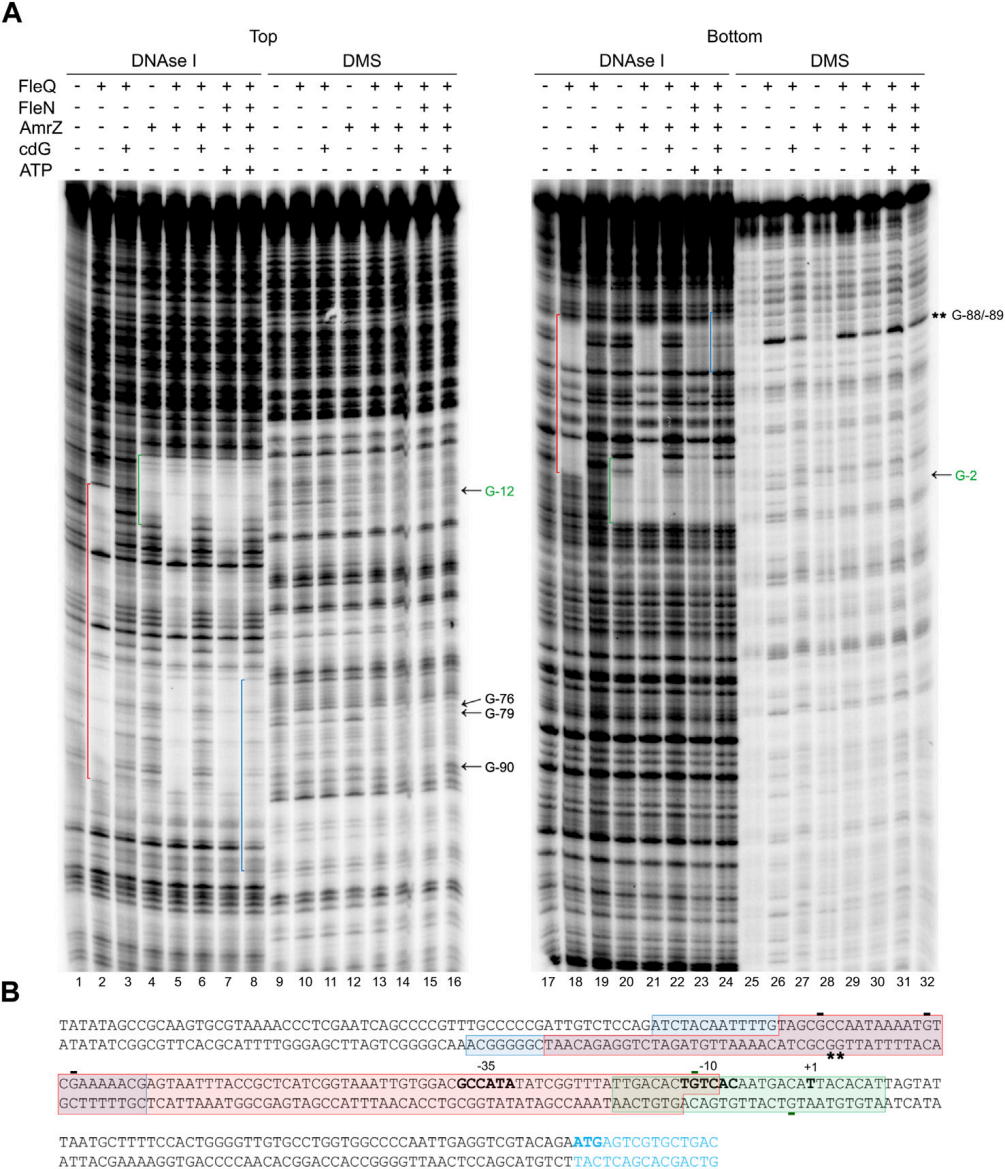
Location of all the regulators binding sites at the *wss* promoter. **(A)** DNAse I and DMS footprint. DNA probes corresponding to the *wssA* upstream region 5’ end-labeled on either the top or the bottom strand were prepared and incubated without (lanes -) and with (+) FleQ and/or FleN, AmrZ, ATP and c-di-GMP. After partial digestion with DNase I or treatment with DMS and partial digestion with piperidine, the DNAs were subjected to urea-PAGE. Nucleotide sequences protected by FleQ and FleQ-FleN in the absence of c-di-GMP (red), by the FleQ-FleN complex in the presence of c-di-GMP (blue) or by AmrZ both in the absence and in the presence of c-di-GMP (green) are indicated. Protected (arrow) and hyperreactive (*) nucleotides are also indicated. **(B)** Simultaneous binding of FleQ, FleQ-FleN and AmrZ at the wss promoter. The boxes indicate the regions protected from DNAse I by FleQ or FleQ-FleN in the absence of c-di-GMP (red), FleQ-FleN in the presence of c-di-GMP (blue) or AmrZ both in the absence and in the presence of c-di-GMP (green) in the top and bottom strands. The -10 and -35 regions and the *wss* transcriptional start site are in bold. *, indicates hyperreactivity and -, protection.

Since AmrZ also regulates the expression of the *wss* promoter (Prada-Ramírez et al., 2016; Pérez-Mendoza et al., 2019), AmrZ was added to the EMSA and footprint reactions to study its effect on the complex formation. The presence of AmrZ in EMSA further retarded the FleQ-DNA and FleQ-FleN (±ATP) complexes, indicating that FleQ, FleN and AmrZ are part of the complex with the wssA1-2 DNA fragment (Figure 5). This was confirmed with the DNase I and DMS footprinting assays (Figure 6). AmrZ bound to the *wssA* promoter protecting the region from −20 to + 8 against DNAse I in both strands, and the G-12 at the top strand and the G-2 at the bottom strand from DMS methylation, as was previously shown (Prada-Ramírez et al., 2016; Pérez-Mendoza et al., 2019). The protection remained the same regardless the presence of FleQ, alone or together with FleN, c-di-GMP, c-di-AMP or ATP, corroborating that AmrZ stays bound to the DNA even when FleQ dissociates (Figure 6 lanes 4-7 and 20-23) or the FleQ/FleN-ATP complex retracts upstream the promoter (Figure 6 lanes 8 and 24).

**FIGURE 7:**
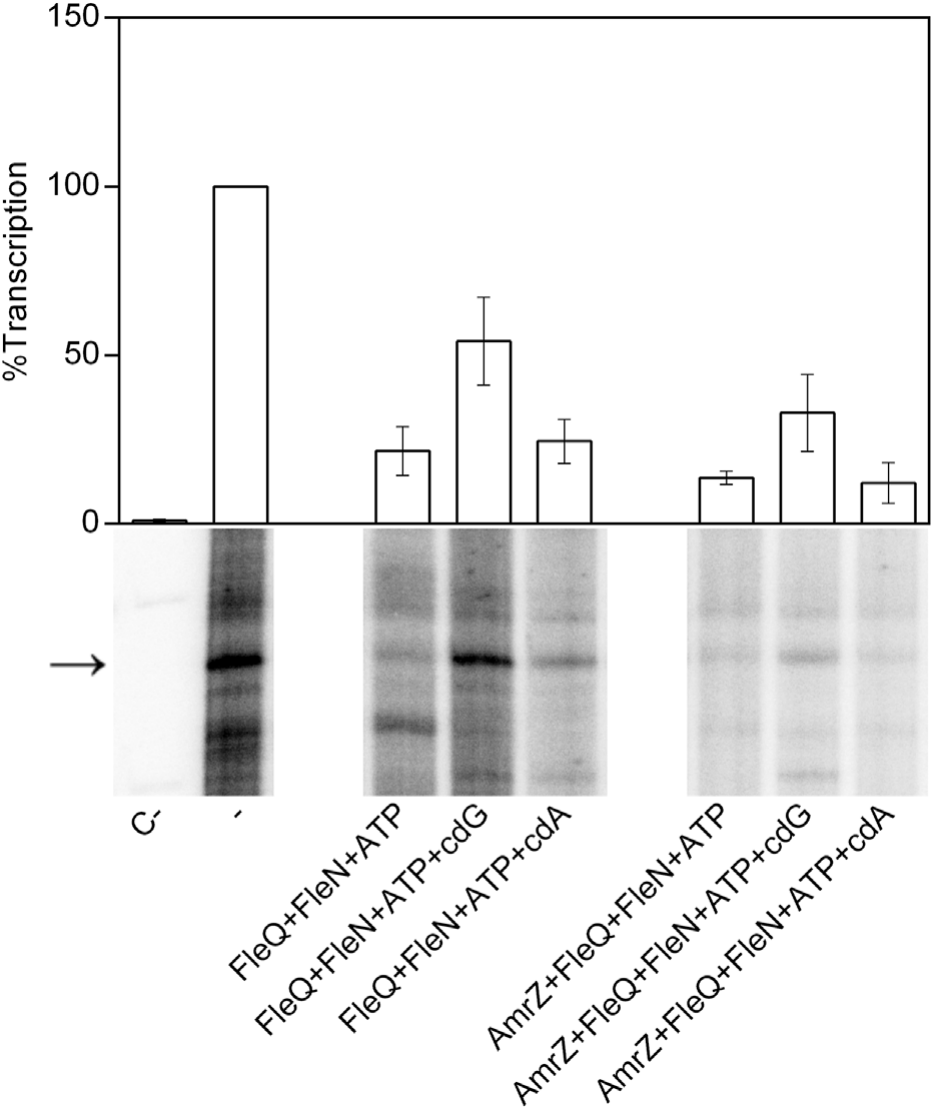
*In vitro* transcription. Multiple round transcription assays were carried out as described in Materials and Methods. The assays were performed in the absence (-) or in the presence (+) of FleQ, FleN or AmrZ. When indicated, ATP, c-di-GMP or c-di-AMP were also added to the reaction. C- indicates a reaction without template DNA. The 232 nucleotide mRNA synthesized from P_*wssA*_ is point out by an arrowhead. The graph shows the average amount of the mRNA produced as percentage of the condition without any protein (-). Error bars correspond to the standard deviation of six different transcription assays.

To check the functionality of the proteins we performed *in vitro* transcription assays with FleQ, FleN and AmrZ both in the absence and in the presence of c-di-GMP and c-di-AMP (Figure 7). We observed that the FleQ-FleN complex repressed (4-fold) the expression of the *wss* operon in the absence of c-di-GMP or in the presence of c-di-AMP. This behaviour is similar to that of FleQ alone (Pérez-Mendoza et al., 2019). The presence of AmrZ further reduced *in vitro* transcription, although the presence of c-di-GMP partly improved it (Figure 7). In summary, all the *in vitro* assays reveal that FleQ/FleN-ATP and AmrZ are able to bind to the DNA at the same time repressing the expression of the *wss* operon under low c-di-GMP levels. When the c-di-GMP levels are high, AmrZ remains bound to the DNA, but the remodelling of the FleQ/FleN-ATP complex on the DNA allows *wss* transcription.

### FleQ binding sites at the *wss* promoter

**FIGURE 8:**
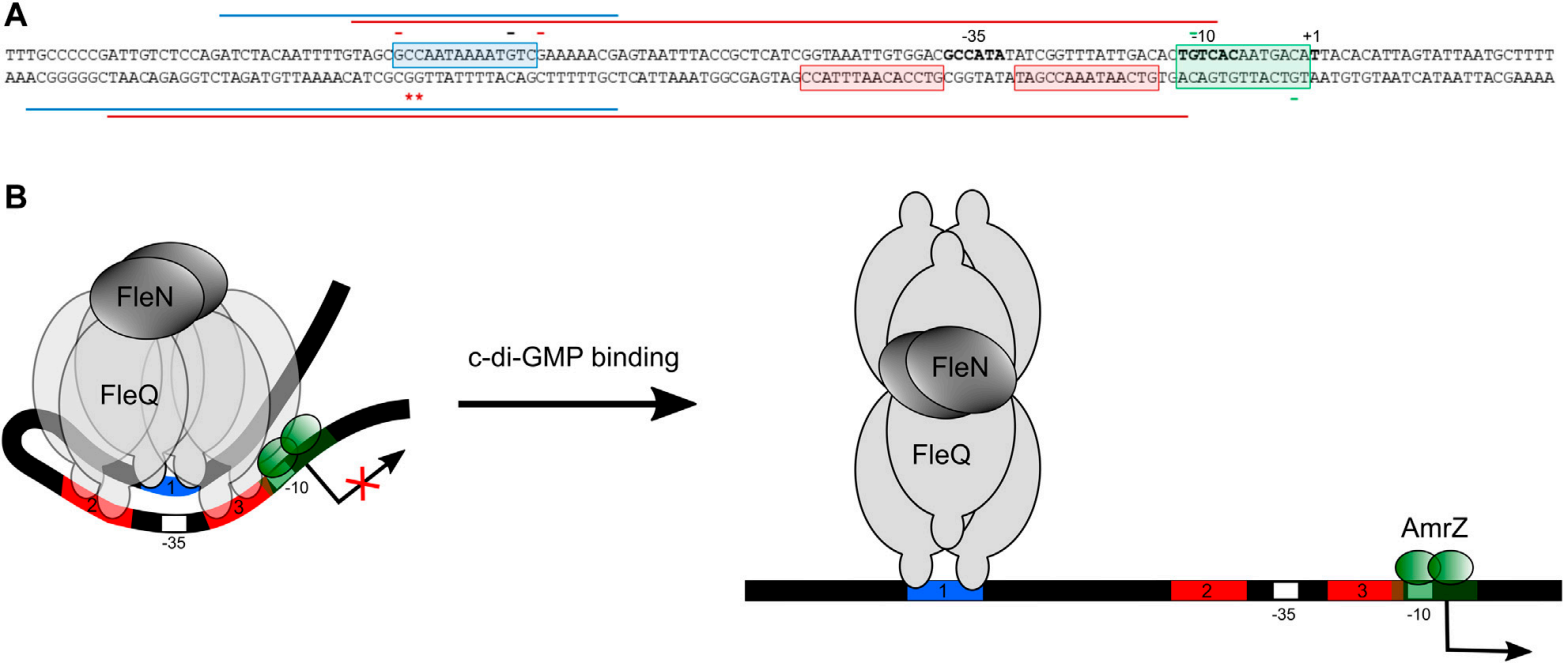
Molecular model of FleQ/FleN-mediated *wss* transcription. **(A)** Summary of *in vitro* and *in silico* results regarding the binding of FleQ, FleN, AmrZ at the *wss* promoter in the absence (red line) and in the presence (blue line) of c-di-GMP. The -10 and -35 regions and the *wss* transcriptional start site are in bold. *, indicates DMS hyperreactivity and -, DMS protection. The red colour shows that the protection or reactivity is detected in the absence of c-di-GMP, the black that the protection takes place both in the absence and in the presence of c-di-GMP, and the green colour indicates that the protection is due to the presence of AmrZ. **(B)**. The diagram depicts a model incorporating our *in vitro* and functional data, and considering previous structural and functional information regarding FleQ from *P. aeruginosa* (Matsuyama et al., 2016).

We examined the Pto DC3000 *wss* promoter region for the presence of FleQ binding sequences and we found three putative sites within the DNAse I protected region (Figure 8) matching the *P. aeruginosa* and *P. putida* sites characterized in previous studies (Jyot et al., 2002; Baraquet et al., 2012; Baraquet and Harwood, 2016; Molina-Henares et al., 2017; Leal-Morales et al., 2022; Nie et al., 2022). Furthermore, those sites (named box 1, 2 and 3) also agreed with the protections and hyperreactivities detected with DMS in the presence of FleQ (Figures 4, 6). FleQ contacts boxes 1, 2 and 3 both on its own and in complex with FleN-ATP. However, after binding c-di-GMP by the FleQ/FleN-ATP complex the protein contacts with the DNA changed and only box 1 was protected (Figures 4, 6).

Additionally, we observed that the sequences located upstream of the *wssA* homologues were well conserved in several *Pseudomonas* strains belonging to the *P. syringae*, *P. fluorescens* and unclassified *Pseudomonas* groups. Particularly, the three FleQ binding sequences and the AmrZ recognition site were highly conserved (Supplementary Figure S3A). It is also remarkable that the sequence of the promoter region and, particularly, the FleQ and AmrZ binding sites, are almost identical in the strains of the *P. syringae* complex that carry the cellulose synthesis operon (Supplementary Figure S3A). From these observations, we can conclude that the FleQ and AmrZ binding sites are present in the *Pseudomonas* species bearing the *wss* cellulose synthesis operon, suggesting that the regulatory mechanism may be also conserved in the *syringae* and *fluorescens* groups.

## Discussion

Cellulose production by Pto DC3000 is a complex process controlled at multiple levels. It has been shown to be post-translationally regulated by c-di-GMP as this second messenger allosterically activates the cellulose synthase WssB by binding to its PilZ domain. It is also transcriptionally controlled by two independent regulators, FleQ and AmrZ (Prada-Ramírez et al., 2016; Pérez-Mendoza et al., 2019). Under physiological levels of c-di-GMP, both regulators bind directly to the promoter region of the *wssABCDEFGHI* biosynthetic operon, inhibiting its expression. However, their behaviours are completely different under high levels of c-di-GMP: AmrZ represses transcription of the *wss* operon independently of intracellular levels of c-di-GMP, whereas FleQ switches from repressing transcription to activating it upon binding c-di-GMP (Pérez-Mendoza et al., 2019).

In *P. aeruginosa*, FleN functions as an antiactivator of FleQ since FleN interaction with FleQ reduces flagellar gene expression (Dasgupta et al., 2000; Dasgupta and Ramphal, 2001; Baraquet and Harwood, 2016; Chanchal et al., 2017). ATP binding by FleN provokes conformational changes that allow dimer formation, which is the functional form of FleN, being the nucleotide binding but not its hydrolysis by FleN necessary to exert its antagonistic effect on FleQ (Chanchal et al., 2017). The FleN dimer binds to FleQ, modifies its structure and inhibits its ATPase activity, but it does not hinder its binding to the DNA (Dasgupta and Ramphal, 2001). Besides to inhibiting FleQ-dependent transcriptional activation of flagellar genes, FleN take part in the regulation of FleQ-dependent biofilm matrix genes in *P. aeruginosa* and *P. putida*, but its effect depends on the promoter (Hickman and Harwood, 2008; Baraquet et al., 2012; Baraquet and Harwood, 2016; Matsuyama et al., 2016; Nie et al., 2017; Navarrete et al., 2019). Deletion of *fleN* led to a decreased expression of biofilm matrix genes, such as *pelA-F*, *pslA-L*, *cdrAB* and PA2440 in *P. aeruginosa* (Hickman and Harwood, 2008; Baraquet and Harwood, 2016). However, *fleN* deletion in *P. putida* decreased the transcription of *lapA*, but increased that of the *bcs* operon (Nie et al., 2017; Navarrete et al., 2019). Furthermore, it has been recently shown that FleQ and FleN were repressors of the *P. putida* T6SS (Nie et al., 2022). We previously showed that Pto DC3000 FleQ was able to bind at the *wss* promoter and repress its expression. Furthermore, c-di-GMP (but no other nucleotides or di-nucleotides) partially disrupted that FleQ/DNA complex facilitating transcription of the *wss* operon (Pérez-Mendoza et al., 2019). Here we have progressed in the understanding of the underlying molecular mechanism. First, *in vivo* experiments show that both FleN and FleQ are required for repression of the *wss* operon under low c-di-GMP and activation under high c-di-GMP (Figure 2). Second, binding of FleN-ATP to FleQ leads to a more stable FleQ/FleN-ATP/DNA complex (Figure 3). Third, binding of c-di-GMP by the FleQ/FleN-ATP/DNA complex stimulates a change in protein conformation that results in repositioning that complex on the DNA (Figure 4) and activation of transcription (Figure 7). Overall, FleN has a prominent role in the regulation of the *wss* operon in Pto DC3000 and this is different from *P. aeruginosa*, where only a small downregulation in *pel* expression was observed in a mutant lacking *fleN* (Hickman and Harwood, 2008).

*In vitro* experiments showed that FleQ can bind on its own to *wss* DNA (Pérez-Mendoza et al., 2019), but disengages from DNA in the presence of c-di-GMP (Figures 3, 5). This behaviour is not observed *in vivo* with the *fleN* mutant; on the contrary it acts like the *fleQ* mutant. Besides, *in vivo* dual regulatory roles of FleQ and FleN are recognizable, since the expression of the cellulose synthesis operon is intermediate and independent of c-di-GMP levels in both the *fleQ* and the *fleN* mutants (Figure 2C). Those strains also produce less cellulose than the wild type when c-di-GMP intracellular levels are raised (Figure 2B), and their colonies are not so wrinkly as the ones of the wild type (Figure 2A). This situation is more consistent with the *in vitro* results showing that the binding of FleN-ATP to FleQ stabilises this protein complex onto the DNA (Figures 3, 5), and binding of c-di-GMP by the FleQ/FleN-ATP/DNA complex stimulates a change in protein conformation without changing its oligomerization status (Figures 3, 5). Surprisingly, this conformational change triggers the shifting of that complex on the DNA (Figure 4) and results in activation of transcription (Figure 7). The *in vivo* consequences of this regulation are complex, given the role of c-di-GMP not only at transcriptional level on the *wss* operon, but also at posttranslational level on the cellulose synthase. As observed before (Pérez-Mendoza et al., 2019), the loss of *fleQ* made the expression of the *wss* operon intermediate and independent of c-di-GMP. Therefore, the *fleQ* mutant produced less cellulose when intracellular levels were raised, and totally lost the wrinkly colony phenotype of the wild type (Figure 2). The expression of the *wss* operon was also intermediate and independent of c-di-GMP in the *fleN* mutant. However, cellulose production was comparable to the wild type in the absence of c-di-GMP, but closer to the *fleQ* mutant at high c-di-GMP. The colonies were smoother than those of the wild type, but not so much as those of the *fleQ* mutant at high c-di GMP (Figure 2). In general, *in trans* expression of *fleN* in the *fleN* mutant resulted in phenotypes more similar to the *fleQ* mutant, probably due to the sequestration of FleQ by FleN, but it did not have a significant effect on the *fleQ* mutant (both under low and high c-di- GMP), or the wild type at high c-di-GMP (Supplementary Figure S2).

We identified three putative FleQ binding sites in the promoter region of the *wss* operon, all of them located upstream of the transcription start site. We found that FleQ and FleQ/FleN-ATP bind at those sites under low c-di-GMP levels, but the binding radically changes with high c-di GMP. This situation is different from the FleQ binding at the *P. aeruginosa cdrAB*, PA2440, and *psl* promoters, which is independent of the presence of c-di-GMP and FleN (Baraquet and Harwood, 2016), and more similar to the binding of FleQ at the *P. putida* KT2440 *lapA*, *bcs* and K1-T6SSpro1 promoters (Nie et al., 2017; Nie et al., 2022). We propose that box 1, the furthest from the Pto DC3000 *wss* transcription start site, is required for activation, whereas the two downstream boxes (2 and 3), which overlap the -35 promoter region, are required for repression. Our model for *wss* expression is that, in the absence of c-di-GMP, FleQ/FleN binds to the three sites at the *wssA* promoter and induces a distortion of DNA, detected as Gs -88 and -89 hyperreactivity when FleQ was present (Figures 4, 6). This most likely impair RNA polymerase binding and repress *wss* transcription, together with AmrZ. Interestingly, the binding of c-di-GMP induces a conformational change in the FleQ/FleN complex, which decreases its affinity for some promoter interaction sites (boxes 2 and 3) and causes it to retract upstream (to box 1). This relieves the DNA distortion, free the -35 promoter region and allows access to the RNA polymerase, leading to activation of *wss* expression. This model is based on previous results with *P. aeruginosa* FleQ. The 3D structures of its AAA+ domain in complex with c-di-GMP together with *in vitro* and *in vivo* functional studies showed that FleQ_Pa_ undergoes a significant conformational change upon c-di-GMP binding, which in turn causes oligomeric rearrangement (Matsuyama et al., 2016). Likewise, we propose that ring-like hexameric FleQ_Pto_ in complex with FleN-ATP binds boxes 1, 2 and 3 at the *wss* promoter, which inhibits transcription initiation (Figure 2C, Figure 7). Although binding of FleQ_Pto_ alone to the DNA and dissociation in the presence of c-di-GMP is detected *in vitro* (Figures 3–6), this does not seem to be relevant *in vivo* (Figure 2). The association of ATP-bound FleN to the FleQ_Pto_/DNA complex promotes c-di-GMP binding, which is consistent with the requirement of FleN for full FleQ/c-di-GMP mediated activation of *wss* transcription (Figure 2C, Figure 7). Furthermore, the oligomerization state of that complex does not change even though it moves to another site in the DNA. Therefore, we hypothesise that a dimer of FleQ_Pto_ trimers in complex with FleN and c-di-GMP may bind to box 1 (Figure 8), similar to what occurs at the *P. aeruginosa pel* promoter (Matsuyama et al., 2016).

It must be mentioned that AmrZ remains bound at the *wss* promoter limiting its expression independently of FleQ, FleN and c-di-GMP levels (Figures 2–7) (Pérez-Mendoza et al., 2019), therefore we cannot rule out that at least part of the regulation mediated by FleQ/FleN and c-di-GMP, takes place through AmrZ. The positive effect of FleQ/FleN-c-di-GMP over *wss* transcription is noteworthy in the wild type, where AmrZ is present (Prada-Ramírez et al., 2016; Pérez-Mendoza et al., 2019). Since AmrZ and FleQ binding sites are adjacent, they could interact when bound to the *wss* promoter, and the conformational changes induced by FleQ/FleN-c-di-GMP in the DNA may disturb the binding and/or the activity of AmrZ as a repressor.

bEBPs from the large AAA + family are specialized transcriptional activators that couple ATP hydrolysis to remodel the promoter-RNA polymerase initiation complex with the σ^54^ factor, converting the initial closed complex to a transcription competent open complex (Gao et al., 2020). FleQ participates in two different transcriptional activation mechanisms, one σ^54^-dependent, as a canonical bEBP, and the other is an unusual σ^70^-dependent one that control EPS production in *P. aeruginosa* (Hickman and Harwood, 2008; Baraquet et al., 2012; Baraquet and Harwood, 2013; Matsuyama et al., 2016). Morevover, FleN further modulates FleQ_Pa_-mediated gene regulation at both class II flagellar genes and *pel* promoters (Hickman and Harwood, 2008; Baraquet et al., 2012; Baraquet and Harwood, 2013). Nevertheless, the FleN_Pa_ effect on flagella expression is much more pronounced, as removal of *fleN* results in cells being multiflagellated, while causing only a small downregulation of *pel* expression (Dasgupta et al., 2000; Hickman and Harwood, 2008). Since the role of FleN in Pto DC3000 cellulose production is so different from that in *P. aeruginosa*, future studies should focus on the molecular function of FleN in flagellar gene expression at Pto DC3000. This regulation should require the FleQ ATPase activity and σ54, that is, a mechanism somehow opposite to that described for the regulation of the *wss* operon. We anticipate that the analysis of FleN and c-di-GMP roles in the expression of flagellar genes in Pto DC3000 will most likely bring some surprises.

## Data Availability

The original contributions presented in the study are included in the article/Supplementary Material, further inquiries can be directed to the corresponding author.

## Supplementary material

Supplementary Figure S1 Pellicle formation by mutant strains of Pto DC3000 under high c-di-GMP levels. The shown strains with pJB3pleD* were grown under aerobic and static conditions at 20°C in 24 multiwell plates for 72 h in MMR supplemented with tetracycline (10 µg/ml). The pellicles formed were disintegrated in 2 ml of deionized water and 8 µl of this suspension was deposited on a clean glass slide and 8 µl of of a 1:1 mixture of 10% KOH (w/v) and calcofluor white stain (Fluka) were added to each sample. A coverslip was deposited on the sample that was immediately examined in an epifluorescence microscope Leica DMI600B (Leica microsystems) using UV light excitation (365 nm). Bars = 10 µm.

Supplementary Figure S2 Colony morphology of the *fleN* mutant and its complemented strain. Representative colony morphology of the indicated strains grown in agar plates supplemented with Congo Red and Calcofluor in the absence (-, pJB3Tc19) and in the presence of *pleD** (+, pJB3pleD*) without *fleN* (-, pBBRN) or with *fleN* expressed *in trans* from a plasmid (+, pBBRN::*fleN*). Five μl of bacterial suspensions at A_660_ = 1.0 were placed on the surface of MMR plates with CR (50 μg/ml, top) or CF (100 μg/ml, bottom) and pictured after incubation at 20°C for 3 days and then at 10°C for 5 days.

Supplementary Figure S3 Multiple sequence alignment of regions upstream of the cellulose synthesis operon found in the *Pseudomonas* genus. **(A)** Multiple sequence alignment of regions upstream of the cellulose synthesis operon found in the *Pseudomonas* genus. The sequences shown are from *Pseudomonas* sp. PAMC 25886 (P_PAMC25886), *P. fluorescens* AU10973 (Pflu_AU10973), *P. trivialis* IHBB745 (Ptriv_IHBB745), *Pseudomonas* sp. TKP (P_TKP), *Pseudomonas* sp. LAMO17WK12:I2 (P_LAMO17WK12:I2), *Pseudomonas* sp. 2-92 (P_2-92), *P. fluorescens* SBW25 (Pflu_SBW25), *P. syringae* CC1557 (Psyr_CC1557), *P. syringae* pv. tomato NYS-T1 (Psyr_tomato_NYS-T1), *P. syringae* pv. tomato DC3000 (Psyr_tomato_DC3000), *P. syringae* BRIP39023 (Psyr_BRIP39023), *P. syringae* pv. coryli NCPPB 4273 (Psyr_coryli_NCPPB4273). **(B)** Multiple sequence alignment of regions upstream of the *wssA* orthologs found in the *P. syringae* group. The sequences shown are from *P. syringae* 31R1 (NZ_LT629769), *P. syringae* UMAF0158 (NZ_CP005970), *P. amygdali* NM002 (NZ_CP020351), *P. savastanoi* NCPPB 3335 (NZ_CP008742), *P. syringae* CFBP 2116 (NZ_LT985192), *P. syringae* isolate CFBP3840 (NZ_LT963409), *P. amygdali* pv. morsprunorum R15244 (NZ_CP026558), *P. avellanae* R2leaf (NZ_CP026562), *P. syringae* isolate CFBP6411 (NZ_LT963408), *P. syringae* pv. actinidiae P155 (NZ_CP032871.1), *P. syringae* pv. actinidiae ICMP 18884 (NZ_CP011972.1), *P. syringae* pv. actinidiae ICMP 18884 (NZ_CM002751.1), *P. syringae* CRAFRU 12.29 (NZ_CP019730), *P. syringae* CRAFRU 14.08 (NZ_CP019732), *P. syringae* NZ-47 (NZ_CP017009), *P. syringae* NZ-45 (NZ_CP017007), *P. syringae* pv. actinidiae ICMP 18884 (NZ_CP011972), *P. syringae* MAFF212063 (NZ_CP024712), *P. syringae* ICMP 9853 (NZ_CP018202), *P. syringae* pv. actinidiae ICMP 9617 (NZ_CM002753), *P. syringae* Ps25 (NZ_CP034558.1), *P. syringae* B13-200 (NZ_CP019871), *P. syringae* pv. tomato delta X (NZ_CP047073.1), *P. syringae* pv. tomato delta IV,IX (NZ_CP047072.1), *P. syringae* pv. tomato delta VI (NZ_CP047071.1), *P. syringae* pv. tomato DC3000 (NC_004578), *P. syringae* isolate CFBP3846 (NZ_LT963402). The putative FleQ binding sites are shown in blue (box 1) and red (boxes 2 and 3), the AmrZ binding site in green, and the putative -10/-35 promoter regions (according to Pto DC3000 transcription start site mapping) are boxed.
