# Supplementary figures and images for: FleQ, FleN and c-di-GMP coordinately regulate cellulose production in Pseudomonas syringae pv. tomato DC3000
Source: Front Mol Biosci. 2023 Mar 27;10:1155579. doi: 10.3389/fmolb.2023.1155579 (PMC10083355; doi:10.3389/fmolb.2023.1155579)

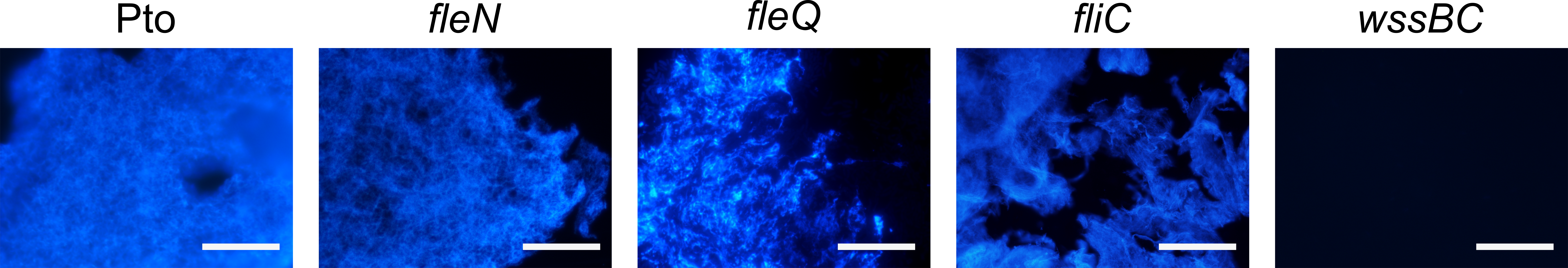

Supplement: Supplementary file 3 [file Image1.TIF]
